# Training for Primary Care Providers: Case Study From Rwandan Cervical Cancer Educate, Screen, and Treat (EST) Program

**DOI:** 10.1200/GO.22.34000

**Published:** 2022-05-05

**Authors:** Callie Weber, Katy Graef, Marc Hagenimana, Elisabeth Mwiza, Justine Kuteesa, Therese Uwamariya, Cassandra Broadwin, Heather Davidson, William Rutagengwa

**Affiliations:** ^1^ BIO Ventures for Global Health (BVGH), Seattle, WA; ^2^ Rwanda Biomedical Centre (RBC), Kigali, Rwanda; ^3^ Nyamata District Hospital (DH), Nyamata, Rwanda; ^4^ GardaWorld, Montreal, Canada

## Abstract

Cervical cancer screening, triage, and treatment programs are expanding across Rwanda's districts. Screening for cervical cancer is needed at the local level, but health center nurses and midwives in the Bugesera district have not received formalized training.

**METHODS:**

The RBC-BVGH Cervical Cancer Educate, Screen, and Treat (EST) Program introduced services in Bugesera district for the first time. In 4-week EST campaign (September 6-October 1], 4,013 women were screened for HPV, 2,056 for VIA, 282 received same-day TA, nine were treated with LEEP at Nyamata DH, and 26 were referred to a Kigali tertiary hospital (Rwanda Military Hospital; RMH) with suspected cervical cancer. Prior to launch of services, providers from 16 health facilities received training (52 nurses/midwives, 17 data managers, 15 lab technicians). The quality and sustainability impact of the training was determined through a pre- and post- course theory assessment (N = 52) and a post-EST survey (N = 15). On the post-EST survey, providers self-ranked their skill confidence across each service before vs. after the campaign on 5-point Likert scale (1 = low and 5 = high).

**RESULTS:**

Following the training program, participants' cervical and breast cancer assessment scores increased by an average of 949% and 405% respectively (*P* < .001). The participants' self-reported confidence in the following techniques also increased: HPV sample collection (206%), visual inspection with acetic acid (187%), thermal ablation (205%), breast examination (54%), and referral of cervical cancer cases (57%). One hundred percent of participants indicated their intent to apply the program learnings in their daily practice yet noted that a refresher training within 3-9 months would be valued.

**CONCLUSION:**

The above results demonstrate that RBC's training programs effectively provide strong theoretical and hands-on training for primary providers. Continued mentorship and refresher trainings are critical for sustainability of high-quality cervical and breast cancer screening, triage, and treatment services in Rwanda.

